# Amphetamines in child medicine: a review of ClinicalTrials.gov

**DOI:** 10.3389/fphar.2023.1280562

**Published:** 2023-10-03

**Authors:** Samer O. Alalalmeh, Omar E. Hegazi, Moyad Shahwan, Fahad S. Alshehri, Ahmed M. Ashour, Alanood S. Algarni, Nasser M. Alorfi

**Affiliations:** ^1^ Center of Medical and Bio-Allied Health Sciences Research, Ajman University, Ajman, United Arab Emirates; ^2^ Department of Clinical Sciences, College of Pharmacy and Health Sciences, Ajman University, Ajman, United Arab Emirates; ^3^ Department of Pharmacology and Toxicology, College of Pharmacy, Umm Al-Qura University, Makkah, Saudi Arabia

**Keywords:** amphetamines, pediatric medicine, ADHD, dependency, developing brain, clinical trials

## Abstract

**Background:** Globally, the use of amphetamines as therapeutic agents in pediatric medicine is a crucial area of concern, especially given the population’s vulnerability.

**Methods:** On 6 August 2023, a search was conducted on ClinicalTrials.gov using “amphetamine” as the keyword. Two independent examiners screened trials against set criteria, including a focus on amphetamine, completion status, an interventional approach, and included children. Ongoing or observational studies were excluded. Data extracted from the qualified trials encompassed primary objectives, participant counts, study duration, and outcomes, with the aim of analyzing children disorders treated by amphetamine.

**Results:** On 6 August 2023, a search of the ClinicalTrials.gov database with the term “amphetamines” identified 179 clinical trials. After extensive exclusion criteria, 19 trials were ultimately selected for analysis. The predominant condition under investigation was attention deficit hyperactivity disorder (ADHD), present in 84.2% of studies. Key study characteristics included: phase 4 trials (36.8%), randomized allocation (63.2%), and the parallel intervention model (42.1%). Masking techniques varied, with no masking in 42.1% of studies, and double and quadruple masking both accounting for 21.1%. Geographically, 78.9% of the studies’ participants were from the United States.

**Conclusion:** This study highlights the notable therapeutic potential of amphetamines in pediatric ADHD populations and emphasizes the importance of recognizing potential side effects and addiction risks. As pharmacogenomics offers the prospect of personalized treatments, there is potential to increase therapeutic efficacy and decrease adverse reactions. It is vital to balance these benefits against the inherent risks, understanding the need for continued research to optimize the use of amphetamines in medicine.

## 1 Introduction

Globally, amphetamines play a significant role in pediatric medicine, both as therapeutic agents and subjects of concern. Their dual role necessitates an in-depth analysis, especially when their application targets vulnerable populations such as children (Meyers et al., 2020). As central nervous system (CNS) stimulants, the effects of amphetamines on a developing brain are both beneficial in specific therapeutic scenarios and potentially harmful if misused (Mental Health Services Administration, 1999). The global prevalence of amphetamine use in 2019 was estimated to be 0.5% in children as an antipsychotic medicine and 12% in pregnant mothers (Ochoa et al., 2023). This extensive utilization, predominantly in the medical treatment of conditions like attention deficit hyperactivity disorder (ADHD), underscores the importance of a thorough review of clinical trials focusing on children and amphetamines (Frölich et al., 2012).

Historically, the recognition of amphetamines dates back to the early 20th century. The medical community began to acknowledge its therapeutic potential for ADHD, a prevalent neurodevelopmental disorder in children (Strohl, 2011). However, the propensity for misuse and consequent dependency, especially in this demographic, raised flags. While the most common amphetamines, amphetamine and methamphetamine, dominate discussions, numerous derivatives and formulations cater specifically for children. Dextroamphetamine is frequently prescribed for pediatric ADHD (Clevel Clin, 2023a), while Adderall, which combines AMPH and dextroamphetamine, stands as a staple in ADHD treatment (Faraone and Biederman, 2002). Lisdexamfetamine metabolizes into dextroamphetamine and remains another crucial ADHD management tool for children (Najib et al., 2020). Although some studies have explored the potential therapeutic applications of MDMA for psychiatric conditions in adults, its effects on children remain largely uncharted and controversial.

The spectrum of amphetamines’ influence on children extends beyond ADHD. For instance, their use in pediatric obsessive-compulsive disorder (OCD) patients has yielded mixed results (OCD Kids, 2023). Similarly, interventions using amphetamines for mood dysregulation disorders in children are treated with caution due to the potential side effects (Parsley et al., 2020). One of the primary concerns is the risk of addiction. A developing brain is susceptible, and the introduction of substances that alter its neurochemistry, especially influencing the dopamine system, requires vigilant monitoring (Berman et al., 2009). As children and adolescents engage with these drugs, even for therapeutic reasons, there is controversial evidence regarding the risk of developing a dependency (Clevel Clin, 2023b).

Another emerging area of research revolves around the long-term effects of amphetamines on the developing brain. Preliminary findings indicate potential structural and functional alterations in specific brain regions with prolonged amphetamine use. These changes, although subtle, might have implications for cognitive functions, emotional regulation, and even social behavior in the long term. With an increasing number of children undergoing amphetamine-based treatments, understanding these long-term implications becomes paramount. Continuous longitudinal studies tracking these children over several years can provide crucial insights into these effects (Reynolds et al., 2015a).

Despite the plethora of individual studies on amphetamines, a consolidated, in-depth examination of clinical trials aimed at children remains a conspicuous gap in the literature. ClinicalTrials.gov, a leading clinical trials database, is a treasure trove of information (Hegazi et al., 2023). However, the data it on amphetamines awaits a rigorous and comprehensive analysis. This not only hinders therapeutic advancements but also affect or reduce understanding of amphetamine’s role in modern medicine. To address this gap, this review offers a comprehensive review of clinical trials from ClinicalTrials.gov on amphetamines.

## 2 Methods

### 2.1 Search strategy

On 6 August 2023, a comprehensive review of ClinicalTrials.gov was conducted using the keyword “amphetamine”. To ensure objectivity, two independent Examiner evaluated the trials returned in the search, based on pre-established eligibility criteria. Trials qualified for inclusion if they predominantly focused on amphetamine, were completed, adopted an interventional approach, and enrolled children. Studies that were still in progress or of an observational nature were excluded. Relevant data were methodically obtained from ClinicalTrials.gov, highlighting vital components that aided the comprehensive analysis of human disorders treated by amphetamine and related substances. This study detailed the primary objectives of each trial and contained essential details such as the number of participants, the length of the study, and the outcomes.

## 3 Results

**FIGURE 1:**
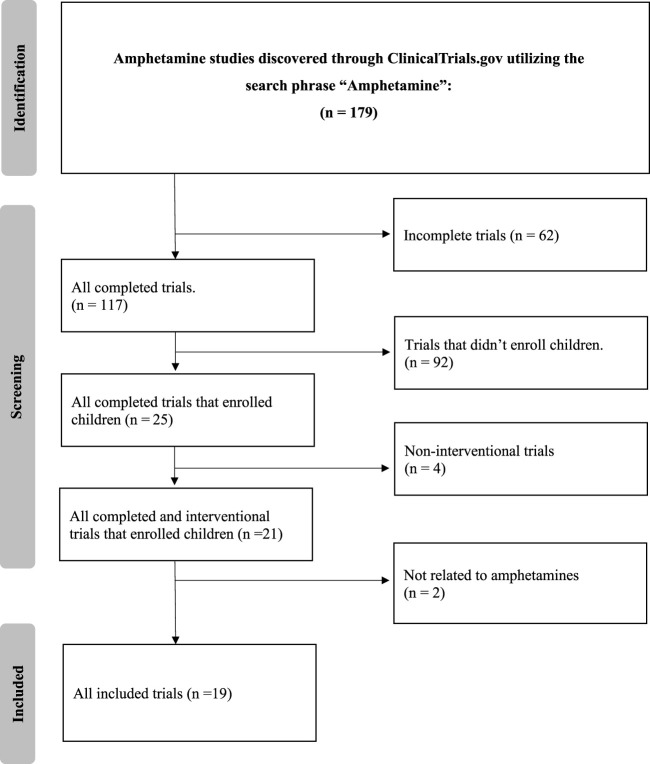
Flow diagram.

The detailed search of the ClinicalTrials.gov database with the term “amphetamine” yielded 179 clinical trials. After applying strict exclusion criteria–incomplete (*n* = 62), non-interventional (*n* = 4), and trials that did not enroll children (*n* = 92)–21 trials were initially suitable. However, two of these did not fully align with our review’s focus, primarily due to their limited emphasis on amphetamine treatments or a focus on unrelated conditions. Thus, 19 trials were finalized for analysis, offering insights into current amphetamine-associated disorder treatments. The selection methodology is illustrated in Figure 1.

### 3.1 Characteristics of included studies

Table 1 provides a detailed breakdown of the various characteristics observed across the 19 trials. The most frequently occurring condition was ADHD, accounting for a significant 84.2%. Three specific sub-groups, each representing 5.3%, were identified: individuals with ADHD coupled with Deficient Emotional Self-Regulation, those with ADHD and Reading Disabilities, and participants with ADHD alongside Conduct Disorder and Oppositional Defiant Disorder.

**TABLE 1:** Clinical trials characteristics.

Characteristics	N (%)
Conditions	
ADHD	16 (84.2)
ADHD and Deficient Emotional Self-Regulation	1 (5.3)
ADHD and Reading Disabilities	1 (5.3)
ADHD, Conduct Disorder, and Oppositional Defiant Disorder	1 (5.3)
Phases	
N/A	2 (10.5)
Phase 1	2 (10.5)
Phase 1 and Phase 2	1 (5.3)
Phase 2	3 (15.8)
Phase 3	4 (21.1)
Phase 4	7 (36.8)
Allocation	
NA	3 (15.8)
Non-randomized	4 (21.1)
Randomized	12 (63.2)
Intervention Model	
Single group	5 (26.3)
Parallel	8 (42.1)
Crossover	6 (31.6)
Masking	
None	8 (42.1)
Single	1 (5.3)
Double	4 (21.1)
Triple	2 (10.5)
Quadruple	4 (21.1)
Locations	
N/A	3 (15.8)
Canada	1 (5.3)
United States	15 (78.9)

### 3.2 The included clinical trials

In terms of the trial phases, Phase four stood out with the highest representation, accounting for 36.8%. Regarding allocation methods, a majority of 63.2% of the trials were randomized. When assessing the intervention model, it was observed that the parallel model was predominant with 42.1% of all studies. In the masking category, 42.1% of the studies had no masking, while both double and quadruple masking were equally represented at 21.1% each. As for geographical distribution, a substantial 78.9% of the participants were based in the United States (Table 2).

**TABLE 2:** Summary of the included trials.

Nct number	Study title	Objectives	Interventions	Primary outcome measures	Secondary outcome measures	Phases	Allocation	Intervention model	Masking	Enrollment	Duration (days)	Locations
NCT01711021	Study to Evaluate Safety and Efficacy of d-Amphetamine Transdermal System vs. Placebo in Children and Adolescents With ADHD	Evaluate the safety and efficacy of d-Amphetamine Transdermal System in treating ADHD in children and adolescents	d-Amphetamine Transdermal System	Assessment of classroom behavior, focusing on attention (SKAMP-A), deportment (SKAMP-D), and work quality through thirteen specific items, measured pre-dose to 12 h post-dose	Alteration in Swanson, Kotkin, Agler, M-Flynn, and Pelham (SKAMP) score’s onset and duration of effect: a 13-item scale assessing classroom behavior. Significant efficacy difference observed between d-ATS and placebo 2 hours post-dose, sustained for up to 12 h (*p* < 0.001)	Phase 2	Randomized	Crossover	Triple	106	151	United States
NCT03610464	Pharmacokinetic Study of DYANAVEL XR (Amphetamine) Extended-release Oral Suspension, in Children Aged 4–5 Years	Study the plasma concentration of amphetamine extended-release oral suspension in 4-5-year-old children with ADHD after a 2.5 mg dose	Amphetamine Extended-Release Suspension [Dyanavel]	Post-dose plasma concentrations of d- and L-amphetamine were measured at specific time intervals ranging from 0 to 28 h	N/A	Phase 4	NA	Single group	None	5	16	United States
NCT00151996	Safety and Tolerability of SPD503 and Psychostimulants in Children and Adolescents Aged 6–17 With attention deficit hyperactivity disorder (ADHD)	Assess the safety of combining SPD503 with psychostimulants for ADHD treatment in children and adolescents aged 6–17	Methylphenidate + SPD503 (Guanfacine hydrochloride) and Amphetamine + SPD503	Using the ADHD Rating Scale-fourth edition (ADHD-RS-IV), a change in the total score was evaluated over 6 weeks. The scale consisted of eighteen items with a scoring range from 0 (absent symptoms) to 54 (severe symptoms)	Improvements noted on CGI-I, CPRS-R, and PGA scores, and CHQ-PF50 increased at 6 weeks, representing enhanced ADHD management and wellbeing. CGI-I and PGA improvements were defined by scores of 1 or 2	Phase 2	Non-randomized	Parallel	None	75	133	N/A
NCT00393042	Sleep and Tolerability Study: Comparing the Effects of Adderall XR and Focalin XR	Compare ADHD treatment responses, sleep effects, and side effects between Adderall XRآ^®^ and Focalin XRآ^®^ in children and adolescents	Dexmethylphenidate and Mixed Amphetamine Salts, ER	Participants’ sleep patterns over 8 weeks were tracked using Actigraphs (AW64 series) in their home settings. Bed and wake times were recorded via sleep logs, and activity data discerned sleep from wakefulness. The impacts of medications like Adderall XR and Focalin XR on sleep were also assessed	ADHD Rating Scale-IV and CGI-S used over 8–10 weeks showed severity of ADHD symptoms and the effects of DAT 1 gene variations. WFIRS indicates functional impairment in children with ADHD.	Phase 3	Randomized	Crossover	Quadruple	77	1,127	United States
NCT01740206	Should Chronic Stimulant Medications be Continued Preoperatively in Patients with Attention Deficit Hyperactivity Disorder (ADHD)	Examine the effects of administering or withholding stimulant medication on surgery day in ADHD patients, assessing blood pressure, heart rate, and medication use	Amphetamine and/or methylphenidate and hold stimulant medication	Heart rate measurement was taken prior to anesthetic induction on Day 1	Pre-anesthetic induction blood pressure measurements were taken. Anxiety was assessed via mYPAS in patients with and without midazolam prior to anesthesia. Higher mYPAS scores signify greater anxiety	N/A	Non-randomized	Single group	None	50	1,125	United States
NCT01986062	Crossover Study to Evaluate the Efficacy of AR11 in Pediatric Patients with ADHD in a Laboratory Classroom Setting	Evaluate ADHD symptom changes in pediatric patients (6–12 years) treated with AR11 or placebo using SKAMP and PERMP assessments	AR11	The SKAMP-combined score, derived from thirteen items with a scoring range of 0 (normal) to 78 (maximum impairment), assessed ADHD symptoms in a laboratory classroom setting, with measurements taken 2 h post-dose	SKAMP-Combined and Subscales assessed ADHD symptoms with 0–6 scales, with higher scores indicating greater impairment. PERMP evaluates ADHD using a 400-question math test, analyzing attempts and accuracy	Phase 4	Randomized	Crossover	Quadruple	97	151	United States
NCT00506727	Analog Classroom Study Comparison of ADDERALL XR With STRATTERA in Children Aged 6–12 With ADHD	Compare ADDERALL XR and STRATTERA’s effects using the SKAMP deportment scale	Mixed amphetamine salts (ADDERALL XR) and Atomoxetine hydrochloride	The change in SKAMP deportment scores from baseline to endpoint was determined over an average of 3 weeks, specifically on Days 7, 14, and 21	After approximately 3 weeks, treatment-emergent adverse events were assessed using PERMP, SKAMP attention scale, CGI, CGIS-P, and Peds QL.	Phase 4	Randomized	Parallel	Double	215	332	United States
NCT02083783	TRI102 in the Treatment of Children with Attention Deficit Hyperactivity Disorder (ADHD)	Determine TRI102’s effectiveness for ADHD treatment in children aged 6–12	TRI102	A change from the baseline in the SKAMP-Combined score, ranging from 0 (normal) to 78 (maximum impairment), was measured approximately 4 h post-dose	PERMP is a math test that evaluated effortful performance and correct answers. The primary efficacy measure was the pre- and post-dose comparison at predetermined timepoints, specifically 4 h after medication intake	Phase 3	Randomized	Parallel	Quadruple	108	275	United States
NCT00507065	Safety and Efficacy of ADDERALL XR in the Treatment of Adolescents Aged 13–17 With Attention Deficit Hyperactivity Disorder (ADHD)	Evaluate ADDERALL XR’s safety and efficacy *versus* placebo in adolescents (age 13–17) with ADHD, considering weight	Mixed salts of a single-entity amphetamine (ADDERALL XR)	Within the cohort weighing ≤75 kg, the change in the ADHD-RS-IV score from the baseline to the conclusion of the study’s double-blind phase was gauged over roughly 4 weeks	Changes in ADHD-RS-IV scores and CGI for ADHD were evaluated from baseline to the double-blind phase’s final visit over approximately 4 weeks. Concurrently, adverse events, labs, physical exams, and ECG results were assessed	Phase 3	Randomized	Parallel	Double	329	301	United States
NCT00889915	Comparing the Effectiveness of New Versus Older Treatments for Attention Deficit Hyperactivity Disorder (The NOTA Study)	Compare the efficacy and tolerability of new *versus* old psychostimulant ADHD medications	Methylphenidate transdermal system, Lisdexamfetamine dimesylate, Osmotic-release oral system methylphenidate (OROS MPH) and Mixed amphetamine salts extended release	Categorizing participants using CGI-E Scale as Responders or Non-responders, based on drug effectiveness and side effects, with scores averaged by week 6	CGI-I and CGI-A scales assessed the improvement and medication acceptability respectively at the participant’s last study visit (latest at Week 6) using a 7-point scale. Both contribute to the primary endpoint (CGI-E)	Phase 4	Randomized	Parallel	None	228	244	United States
NCT03327402	Safety, Tolerability and Pharmacokinetics of SHP465 in Children Aged 4–5 Years with attention deficit hyperactivity disorder (ADHD)	Study the PK, safety, and tolerability of multiple daily 6.25 mg doses of SHP465 in 4-5-year-old children with ADHD.	SHP465	Plasma concentrations of d-Amphetamine and l-Amphetamine were evaluated using rich sampling collection for metrics including Cmax, Tmax, Ctrough, AUC, Lambda z, CL/F, t1/2, and Vss/F during specified times. Vital signs, ECG, height, weight, and laboratory results were monitored. Sleep was assessed using PSQ and CSHQ, and suicidal ideation through C-SSRS during the study, noting that the values may not reflect actual precision	Plasma concentrations of d-amphetamine and l-amphetamine were assessed at 12-, 16-, and 24-h post-dose using a detailed sampling method in Week 4. The area under the concentration-time curve (AUC) for these substances was evaluated between 5 and 12, 12–16, and 16–24 h post-dose	Phase 1	NA	Single group	None	24	206	United States
NCT00557011	NRP104, Adderall XR or Placebo in Children Aged 6–12 Years With ADHD	Assess NRP104 and Adderall XR’s efficacy and safety compared to placebo in 6–12-year-old children with ADHD.	NRP104 and Adderall XR	Assessed SKAMP-DS scores over a week-long treatment evaluation period	At 1 week, the SKAMP Attention Scale, PERMP attempted score, PERMP correct score, and CGI scores were evaluated, alongside treatment emergent adverse events and the PK profile and PK/PD relationship of NRP104 after multiple doses	Phase 2	Randomized	Crossover	Quadruple	52	91	N/A
NCT01133847	Interventions for Children with Attention and Reading Disorders	Compare attention and reading outcomes in children with ADHD and RD under three treatments: ADHD-only, RD-only, and combined treatments	Methylphenidate, Mixed Salt Amphetamine, Atomoxetine and Guanfacine	The SNAP assesses ADHD symptoms in children through parent and teacher ratings on a four-point Likert scale, showing robust reliability. The WIAT-III measures academic achievement with standardized scores. Both were evaluated post 16-week treatment and at follow-up	Post 16-week treatment, evaluations included WIAT-III, DIBELS ORF, TOWRE, and TOSREC, measuring reading comprehension, oral and silent fluency, and word efficiency	Phase 4	Randomized	Parallel	Single	222	1,673	United States
NCT03088267	Dyanavelآ^®^ XR Extended-Release Oral Suspension in the Treatment of Children With ADHD: A Laboratory School Study	Assess DYANAVEL XR’s efficacy and safety for ADHD treatment in children aged 6–12	Amphetamine extended-release oral suspension, 2.5 mg/mL extended-release oral suspension	Assessing ADHD-related classroom impairment, the SKAMP-C scale measures academic tasks, rule adherence, and social interactions. A decrease from baseline to 30 min post-dose indicates improvement with treatment	The PERMP-C Score, assessing academic productivity in school children, was evaluated for changes from pre-dose at 30 min and at 3 h post-dose. A numerical increase indicated an improvement	Phase 3	Randomized	Crossover	Double	18	261	United States
NCT01886469	A Phase II, Adaptive Trial Design Examining the Pharmacokinetic and Pharmacodynamic Effects of Modified Release Amphetamine (HLD100, Formulations B, C and E)) in Adolescents and Children with attention deficit hyperactivity disorder (ADHD)	Study HLD100s absorption rates in adolescents and children with ADHD.	HLD100-B, HLD100-C, and HLD100-E	Measuring d-amphetamine’s absorption rate and extent using AUC0-tz, AUC0-∞, Cmax, Tmax, absorption lag time, λz, and t1/2elim over a 48-h period	Safety aspects, including adverse events, ECG, lab parameters, and physical examinations, were assessed within a 48-h timeframe	Phase 1 & Phase 2	Non-randomized	Parallel	None	22	62	Canada
NCT00228046	Medication Strategies for Treating Aggressive Behavior in Youth with Attention Deficit Hyperactivity Disorder	Examine the effectiveness of combining divalproex sodium with stimulants to reduce aggression in ADHD children	Divalproex Sodium, Methylphenidate, Dextroamphetamine, and Mixed Amphetamine Salts	After 8 weeks of treatment, aggression was assessed using the Overt Aggression Scale, and ADHD symptom improvement was gauged via the Clinical Global Improvement Scale and ADHD Rating Scale	N/A	Phase 4	Randomized	Parallel	Double	40	1,277	United States
NCT00712699	Effectiveness of an Extended-Release Stimulant Medication in Treating Preschool Children With ADHD	Evaluate extended-release mixed amphetamine salts in preschool ADHD treatment	Sequence 1: XR-MAS then placebo. Sequence 2: Placebo then XR-MAS	Weekly assessment for 6 weeks on the Composite Parent and Teacher Conners Rating Scale Score and tolerance of extended-release mixed amphetamine salts	The Clinical Global Impression-Improvement Score was assessed weekly over a 6-week period	N/A	Randomized	Crossover	Triple	27	791	N/A
NCT02578030	Pharmacokinetic Study in Children and Adolescents Aged 6–17 Years Who Have Been Diagnosed With ADHD	Profile the pharmacokinetics of SHP465 in children and adolescents aged 6–17 with ADHD.	SHP465 12.5 mg and SHP465 25 mg	Outcome measures assessed Cmax, Tmax, AUC0-infinity, AUClast, t½, CL/F, and Vz/F for both d-amphetamine and l-amphetamine in plasma at specified post-dose intervals	Post-treatment adverse events, including symptoms, disease, vital sign changes, ECG, lab test anomalies, and C-SSRS-measured suicidal behaviors or ideation, were observed for up to 72 h post-dose	Phase 1	Non-randomized	Single group	None	27	17	United States
NCT02204410	Omega-3 Supplementation to ADHD Medication in Children	Assess Omega-3 fatty acids’ effectiveness for DESR in ADHD children and adolescents aged 6–17 currently on ADHD medication	Omega-3 Fatty Acid and ADHD Medication	The Emotional Control Subscale of the BRIEF-Parent, assessing a child’s emotional response modulation, and the CGI Improvement for Deficient Emotional Self-Regulation, a clinician-rated 7-point scale, both evaluated at baseline and 12 weeks	N/A	Phase 4	NA	Single group	None	21	762	United States

### 3.3 General overview, history, and classification

Amphetamines are classified as potent sympathomimetic agents with valuable therapeutic uses. Chemically, they share a great similarity with the body’s innate catecholamines, specifically dopamine and norepinephrine (Heal et al., 2013). First synthesized in 1887 by Lazăr Edeleanu in Germany, their significance in the field of pharmacology was not acknowledged until the 1950s, when they emerged as a potential treatment for certain behavioral disorders (Edeleanu, 1887). The term “amphetamine” designates a particular compound, predominantly present in two enantiomers: levoamphetamine and dextroamphetamine. These mirror-image molecules, while structurally related, vary in terms of their potency and pharmacological effects (Heal et al., 2013). Noteworthy derivatives such as methamphetamine and para-methoxyamphetamine (PMA) also belong to the amphetamine family, and each has a unique set of characteristics (Gough et al., 2002).

### 3.4 Mechanism of action and medical uses

Amphetamines act by facilitate the release of catecholamines, especially dopamine, from presynaptic neurons while simultaneously blocking their reuptake. This dual action increases their concentration within the synaptic cleft, thereby extending neurotransmission durations (Heal et al., 2013). Elevated dopamine concentrations in the brain’s mesolimbic dopamine system not only lead to feelings of euphoria but also heighten the risk of addiction, further underscoring the potential for amphetamine dependence (Adinoff, 2004; National Institute on Drug Abuse, 2007). On a related note, increased norepinephrine levels within the CNS are correlated with heightened alertness and arousal (Berridge, 2008). When prescribed and monitored by medical professionals, amphetamines serve as effective treatments for conditions such as ADHD, narcolepsy, and, in some cases, treatment-resistant depression. Their therapeutic applications include enhanced wakefulness, improved cognitive control, reduced fatigue, and mood elevation (Stotz et al., 1999; Berman et al., 2009; Ng and O’Brien, 2009).

### 3.5 Side effects, concerns, and legal implications

Extended and consistent misuse of amphetamines can result in a range of adverse side effects, including inhibited growth, heightened jitteriness, feelings of nausea, and diminished visual clarity (Berman et al., 2009; Craig et al., 2015; Richardson et al., 2017). Long-term abuse escalates the risk of complications such as pronounced dental deterioration commonly referred to as “meth mouth”, significant weight loss, persistent skin lesions, an increased dependency, and a powerful addiction (Craig et al., 2015; Nida, 2019). When an addiction takes hold, users often find themselves developing a tolerance. This can lead to overwhelming cravings, pronounced withdrawal symptoms, and a relentless cycle of consumption, even when faced with detrimental repercussions (Leith and Kuczenski, 1981). In recognition of their substantial potential for abuse, amphetamines are stringently regulated on a global scale. Within the United States, these drugs are categorized as Schedule II controlled substances (Deadiversion, 2023). Due to their elevated risk of abuse in the course of their medical use, their prescription and distribution are heavily restricted. Comparable regulations are implemented internationally, and those found accountable of unauthorized distribution or trafficking can expect severe legal consequences (Marandure et al., 2023).

### 3.6 Pharmacokinetics

It is crucial to comprehend the pharmacokinetics of amphetamines, not only in order to optimize their therapeutic usage but also to detect their potential for misuse. Once taken orally, these compounds are rapidly absorbed from the gastrointestinal system, reaching their peak concentration in the bloodstream approximately 2, 3 h after ingestion. It is worth noting that the liver plays a central role in their metabolism (Berman et al., 2009; National Institute of Diabetes and Digestive and Kidney Diseases, 2012).

### 3.7 Detailed compound breakdown

#### 3.7.1 Amphetamine

**FIGURE 2:**
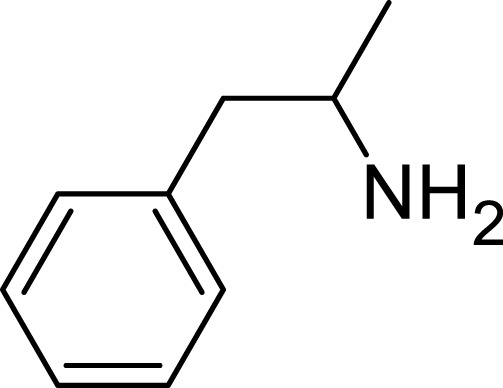
Amphetamine structure.

Amphetamine (C_9_H_13_N), a derivative of the phenethylamine family, manifests in two distinct enantiomeric forms: levoamphetamine and dextroamphetamine. Notably, dextroamphetamine demonstrates a heightened potency in stimulating the CNS (Berman et al., 2009; Zanda et al., 2017; Losacker et al., 2022). At the neurochemical level, the primary mechanism of action for amphetamine involves the elevation of neurotransmitter levels within the synaptic junctions. This elevation arises from the compound’s disruption of vesicular monoamine transporters, subsequently triggering the release of key neurotransmitters–namely, dopamine, norepinephrine, and serotonin–into the synaptic space (Heal et al., 2013). In addition to this, amphetamine has the capability to delay the reuptake of these neurotransmitters, thereby further amplifying their presence and concentration within the synapse (dela Peña et al., 2015) (Figure 2).

#### 3.7.2 Dextroamphetamine

**FIGURE 3:**
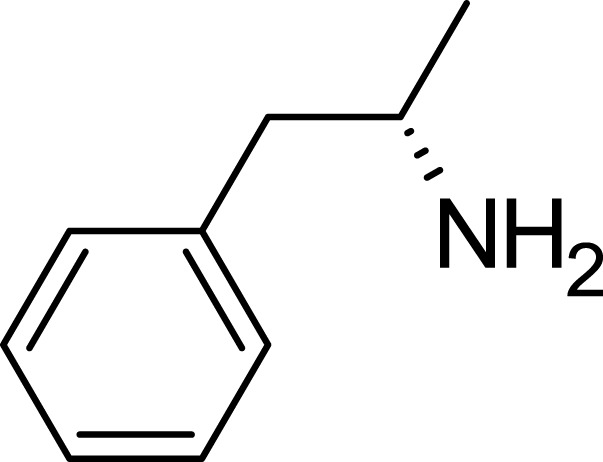
Dextroamphetamine structure.

Dextroamphetamine, chemically represented as C_9_H_13_N, stands out as one of the two active enantiomers of amphetamine. This prescription medication plays a pivotal role in treating conditions such as ADHD and narcolepsy (Sharbaf Shoar et al., 2023). A notable mention is Adderall, a widely recognized medication for ADHD, which comprises four distinct amphetamine salts. However, dextroamphetamine predominates in its composition (Kolar et al., 2008). Distinguished as (S)-amphetamine, dextroamphetamine is an optically active isomer of the parent compound, amphetamine (Heal et al., 2013). With a chemical structure denoted by C9H13N, its chiral center permits the molecule to manifest in two distinct enantiomeric forms. The “dextro” prefix not only signifies its specific structural configuration but also underscores its capability to rotate plane-polarized light toward the right (Figure 3).

#### 3.7.3 Levoamphetamine

**FIGURE 4:**
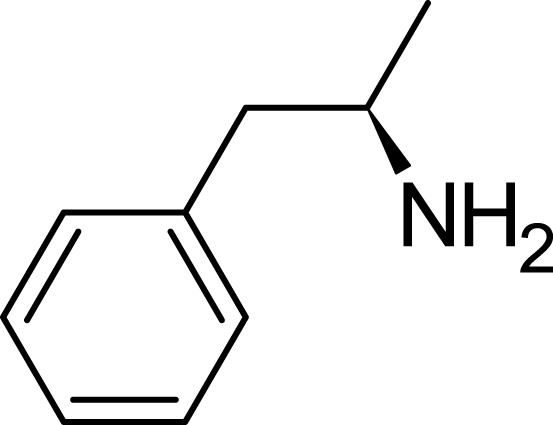
Levoamphetamine structure.

Levoamphetamine, often overshadowed by its more renowned counterpart, dextroamphetamine, is another enantiomer of amphetamine. Unlike dextroamphetamine which primarily has central stimulant effects, levoamphetamine exhibits a stronger peripheral stimulant action. This means that it might lead to more pronounced cardiovascular effects, such as an increased heart rate (Heal et al., 2013; Chen et al., 2023). Levoamphetamine is present in medications such as Adderall, but in reduced quantities compared to dextroamphetamine (Sontheimer and Sontheimer, 2021). Structurally, while both dextroamphetamine and levoamphetamine share the same chemical formula (C9H13N), they differ in the spatial orientation of their atoms (Gough et al., 2002; Heal et al., 2013). This distinction, known as chirality, is reflected in levoamphetamine’s name; the prefix “levo” denotes that it rotates plane-polarized light to the left. Although both compounds share many characteristics, this chiral difference gives levoamphetamine a unique pharmacological profile. Specifically, its interactions with neurotransmitter systems deviate from those of dextroamphetamine due to this structural variation (Goodwin et al., 2009; Heal et al., 2013) (Figure 4).

#### 3.7.4 Methamphetamine

Methamphetamine, a potent derivative of amphetamine (Hall et al., 2008), is known for its heightened effects on the CNS. Although it has legitimate medical uses–available in prescription form as Desoxyn to treat certain cases of ADHD and obesity–it is more notoriously associated with the illicit drug “crystal meth” (Miller et al., 2021). A distinguishing feature of methamphetamine is its elevated lipid solubility, enabling it to penetrate the blood-brain barrier with greater concentration than other amphetamines (Jan et al., 2012; Kirkpatrick et al., 2012). This characteristic significantly escalates its potential for abuse and addiction, particularly when in its crystalline form.

**FIGURE 5:**
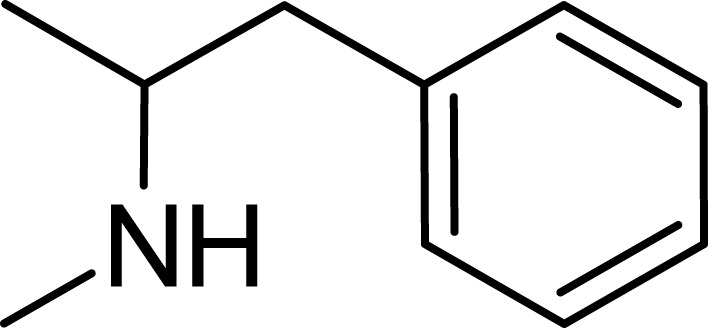
Methamphetamine structure.

From a chemical perspective, methamphetamine, also known as N-methylamphetamine, stands apart from amphetamine due to the inclusion of a methyl group on its nitrogen atom (Kirkpatrick et al., 2012). Its molecular formula reads as C_10_H_15_N. While this modification might seem subtle, it has a profound influence on the compound’s pharmacokinetics. The added methyl group enhances the compound’s lipid solubility, facilitating its rapid movement across the blood-brain barrier (Kirkpatrick et al., 2012). Moreover, methamphetamine exists as two enantiomers: dextromethamphetamine and levomethamphetamine (West et al., 2012) (Figure 5).

#### 3.7.5 Lisdexamfetamine

**FIGURE 6:**
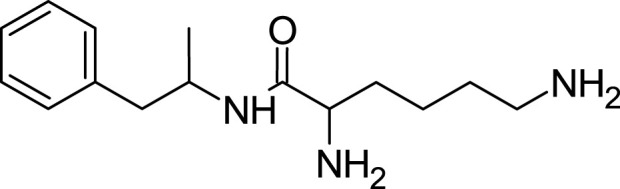
Lisdexamfetamine structure.

Lisdexamfetamine is a prodrug of dextroamphetamine. It therefore remains inactive upon ingestion, and needs to be activated by metabolic conversion to manifest its active form (Cho and Yoon, 2018). What sets Lisdexamfetamine apart is its intrinsic extended-release mechanism due to its prodrug nature. This not only ensures a more prolonged therapeutic effect but also mitigates its potential for misuse. The reason behind this is its more gradual onset of action, compared to other immediate-release amphetamine formulations. Chemically, Lisdexamfetamine is an amide conjugate, formulated by combining dextroamphetamine with the essential amino acid lysine (Mrazek et al., 2009; Levine and Swanson, 2023). Its chemical formula is C_15_H_25_N_3_O (Figure 6).

#### 3.7.6 Para-methoxyamphetamine

**FIGURE 7:**
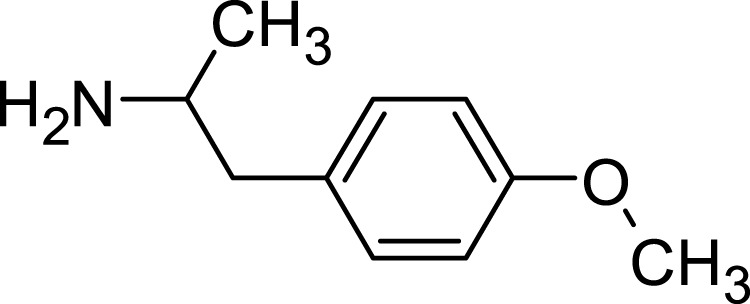
Para-methoxyamphetamine structure.

PMA, or para-Methoxyamphetamine, is a synthetic compound that shares a structural resemblance to amphetamines but stands apart due to its unique pharmacological profile (Richter et al., 2019). Although occasionally mistaken for MDMA (commonly known as “Ecstasy”), PMA’s effects can be considerably more toxic. This compound specifically interacts with the brain’s serotonin receptors to produce psychedelic experiences. However, one of its alarming side effects is a potentially hazardous surge in body temperature, making it significantly more perilous than many of its amphetamine counterparts (Freezer et al., 2005). Chemically, while PMA retains the foundational structure of amphetamines, it differentiates itself with an additional methoxy group situated at the para position of the phenyl ring. Its molecular formula is represented as C_10_H_15_NO (Figure 7).

### 3.8 Common conditions managed by amphetamines

#### 3.8.1 Attention deficit hyperactivity disorder

Amphetamines are recognized for their wide-ranging applications, but are particularly notable for their role in treating ADHD (Frölich et al., 2012). ADHD is a complex neurodevelopmental disorder, typified by enduring patterns of inattention, heightened hyperactivity, and pronounced impulsivity (Magnus et al., 2023). The precise origins and causes of ADHD have yet to be definitively ascertained; however, the prevailing scientific consensus shows that imbalances in neurochemicals, especially within dopamine pathways, significantly influence its manifestation (Blum et al., 2008; Wilens and Spencer, 2010a). Medications formulated with amphetamines such as Adderall, which combines amphetamine and dextroamphetamine, and Evekeo, aim to modulate these neurotransmitter concentrations. As a result, they effectively mitigate the predominant symptoms of the disorder (Frölich et al., 2012).

#### 3.8.2 Narcolepsy

Narcolepsy is a long-term sleep disorder marked by an intense and uncontrollable propensity for daytime sleepiness involuntary sleep episodes. People with this condition may find themselves inadvertently dozing off during daily activities, which presents not only an inconvenience but also poses potential safety risks. While the precise mechanisms underpinning narcolepsy remain a topic of ongoing research, many cases have been linked to a deficiency in the neuropeptide hypocretin (Akintomide and Rickards, 2011). Due to their stimulatory effects, amphetamines have demonstrated efficacy in mitigating the debilitating daytime drowsiness symptomatic of narcolepsy. Consequently, medications such as Adderall are frequently incorporated into treatment regimen for the disorder (Turner, 2019; Barker et al., 2020).

#### 3.8.3 Obesity and weight management

Historically, Phentermine, an amphetamine derivative, was prescribed as an anorectic or appetite suppressant to assist with weight reduction (Coulter et al., 2018). Its stimulant properties have the potential to accelerate metabolism and reduce appetite. In the mid-20th century, drugs such as Benzedrine gained prominence for their weight management benefits. However, increasing concerns regarding the potential for misuse, adverse cardiovascular implications, and other side effects precipitated a decrease in their utilization for this objective. It is crucial to underscore that in contemporary medical practice, the prescription of amphetamines solely for weight loss is not commonly endorsed due to these aforementioned concerns (Abenhaim et al., 1996).

#### 3.8.4 Treatment-resistant depression (TRD)

TRD is characterized by major depressive episodes that fail to show sufficient improvement, even after the administration of at least two distinct antidepressant regimens. Recognizing the mood-elevating and energy-boosting properties of amphetamines, researchers have explored their potential as adjunctive treatments for TRD. While certain studies have yielded promising outcomes, the application of amphetamines in this specific scenario is still off-label. It is essential for more comprehensive research to be conducted to firmly determine both their safety and efficacy in treating TRD (Stotz et al., 1999).

#### 3.8.5 Cognitive enhancement and fatigue management

In specific situations, amphetamines have been employed off-label as tools for cognitive augmentation and combating fatigue. The underlying intent is to bolster alertness, sharpen concentration, and enhance overall performance during extended durations of wakefulness or in instances of sleep deprivation. Nonetheless, the repercussions of prolonged use remain inadequately researched, accompanied by legitimate concerns surrounding the potential for misuse and subsequent dependency (Ricci, 2020). With cognitive deterioration being a significant issue in the senior demographic, there has been a growing interest in evaluating the efficacy of stimulants, including amphetamines, in amplifying cognitive abilities in older adults who do not suffer from dementia. Initial research hints at possible advantages in tasks demanding attention and memory, yet the long-term safety and effectiveness for this demographic are still to be conclusively determined (Bagot and Kaminer, 2014).

#### 3.8.6 Traumatic brain injury (TBI)

After experiencing TBI, many patients may face several challenges, such as cognitive impairments, diminished alertness, and delayed processing speeds. Emerging studies have proposed that amphetamines could potentially accelerate recovery and improve cognitive outcomes for these individuals. The postulated mechanism behind this effect is the drug’s capacity to boost synaptic transmission and amplify neural plasticity, which might bolster the brain’s innate healing mechanisms. Nevertheless, the role of amphetamines in the rehabilitation of TBI is a topic under active investigation, and concrete conclusions regarding their effectiveness have yet to be firmly established (Hornstein et al., 1996; Coris et al., 2021).

## 4 Discussion

It is important to have a comprehensive grasp of the underlying mechanisms, applications, potential adverse effects, and the historical trajectories of drugs is pivotal to ascertaining their suitability and safety for diverse medical conditions. Our meticulous examination of clinical trials centered on amphetamines, strengthened by a profound study of their historical trajectory and present-day status, shows their multifaceted roles, advantages, and associated risks.

From our extensive analysis, it is apparent that the body of clinical trials on ADHD is coherent, reflecting amphetamines’ historical significance and contemporary relevance in managing the disorder. Patients with ADHD, a disorder delineated by its hallmark symptoms of inattention, hyperactivity, and impulsivity, have experienced transformative treatment outcomes with amphetamine-based therapies (Singh et al., 2015). These medications, through their influential role in altering neurotransmitter concentrations, predominantly dopamine, present a compelling strategy for targeting the fundamental symptoms of the disorder (Gough et al., 2002; Heal et al., 2013). Recognized as a neurodevelopmental anomaly that predominantly surfaces during formative years (Wilens and Spencer, 2010b), studies have underscored the pivotal nature of amphetamine-centric interventions for this patient cohort. Notwithstanding their pronounced effectiveness in symptom alleviation, concerns related to side effects like impeded growth demand vigilant scrutiny and periodic oversight, especially among children (Richardson et al., 2017). Moreover, the prospective repercussions on growing neural trajectories and the consequent implications for long-term cognitive capacities call for thorough, sustained investigations (Berman et al., 2009; Reynolds et al., 2015b).

Turning to narcolepsy, a persistent sleep affliction, the therapeutic approach with amphetamines support the fact of determining drug pharmacodynamics in depth. Controlling the stimulative effects of amphetamines, lethargy during the day can be reduced for patients. However, it remains imperative to consistently balance the medicinal gains against any potential adverse outcomes or addiction susceptibility. The assessment of amphetamines across diverse conditions such as obesity, TRD, cognitive augmentation, and TBI unveils the expansive therapeutic potential of these molecules. Yet, as evidenced in the context of obesity management, the evolving landscape of medical protocols and burgeoning knowledge can recalibrate drug adoption trends. The diminished preference for amphetamines in weight management due to concerns over potential misuse and adverse reactions accentuates the necessity for constant evaluation, supervision, and recalibration of clinical directives.

Furthermore, while the therapeutic promise of amphetamines for addressing TRD and enhancing cognition, particularly among the geriatric populace or those with post-traumatic brain injuries, is captivating, it necessitates prudence. The off-label deployment of medications frequently navigates the unclear of clinical practice, making it vital to understand risks against the prospective benefits. A detailed dissection of specific amphetamine derivatives shows the intricate distinctions between them, highlighting that even minor chemical alterations can exert significant effects on pharmacokinetics and pharmacodynamics. This layered understanding can adeptly steer drug choices in specific clinical contexts.

### 4.1 Future directions

Future investigations into amphetamines should place a high emphasis on pediatric-focused trials, particularly in light of the rising prescriptions for children with ADHD. Alongside this, it is crucial to delve deeper into the intricate mechanisms underpinning addiction, while simultaneously developing robust prevention strategies. Additionally, considering the pharmacogenomics may offer tailored dosing and more predictable patient outcomes. Moreover, there exists a significant opportunity to innovate in the domain of extended-release formulations, aiming to affect an optimal balance between maximizing therapeutic advantages and minimizing the potential for misuse.

## 5 Conclusion

This study has underscored both the significant therapeutic potential of amphetamines, especially evident in pediatric ADHD populations, and the crucial need for awareness of their potential side effects and addiction risks. As the landscape of medicine expands, innovative formulations and broader therapeutic applications are emerging, with the exciting prospect of pharmacogenomics potentially redefining individualized treatments. This promises to reduce adverse reactions and bolster therapeutic efficacy. Balancing these benefits with the inherent risks remains paramount. Therefore, ongoing research is crucial in order to further understand amphetamines’ broad applications and to navigate the balance between their benefits and risks.

## Data Availability

The original contributions presented in the study are included in the article/Supplementary material, further inquiries can be directed to the corresponding author.
